# Will the 10-year fishing ban save the Yangtze River?

**DOI:** 10.1093/nsr/nwag299

**Published:** 2026-05-21

**Authors:** Weijie Zhao

## Abstract

The 10-Year Fishing Ban (2021-2030) prohibits productive fishing in the Yangtze River. While various indicators rebounded in its first half, recovering the entire river ecosystem requires more than a fishing ban.

**Figure:**
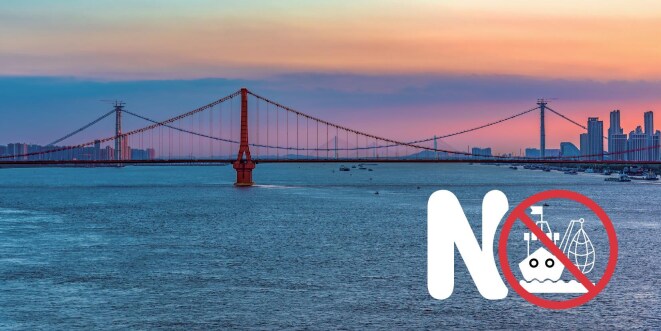
The Wuhan Yingwuzhou Yangtze River Bridge.

Figure credit: Shutterstock-1707805 966-QQQQQQQT.

In the autumn of 2025, an elderly man was fishing by the river. This is the Yangtze River, the longest river in China and in Asia, the third longest worldwide, and the mother river of Chinese civilization. Suddenly, a buzzing sound came from afar. The old man looked up and saw a drone flying closer, finally hovering above his head and speaking: ‘This is a fishing-ban control area along the Yangtze River. Your act violates relevant laws and regulations. Please stop immediately.’ The old man put away his fishing gear, and the drone turned back.

On 1 January 2021, China officially started the 10-year fishing ban in the Yangtze River Basin, prohibiting productive fishing of natural fishery resources in key waters of the Yangtze River for a decade. By 2023, 111 000 fishing boats had been withdrawn from fishing, involving 231 000 fishermen. By the end of the third quarter of 2025, 142 000 retired fishermen with working ability and willingness had all been re-employed in other industries; 220 000 eligible retired fishermen had participated in pension insurance, and 60 000 of them had started receiving pensions.

The 10-year fishing ban features a long time duration and wide space coverage. Coordinated by the Ministry of Agriculture and Rural Affairs, a number of governmental departments and 15 provinces, autonomous regions, or provincial-level cities are involved. It requires not only sustained massive investment in human, material, and technological resources, but also scientific design and management.

Take ‘recreational fishing’ as an example. It is not completely banned. Chongqing, a city on the upper Yangtze River, and many other areas have issued their *Measures for the Administration of Recreational Fishing in Fishing-Ban Waters*, stipulating that recreational fishing is allowed outside key fishing-ban zones and periods, with strict limits on permitted fishing tools, and methods, as well as the species, quantity, and size of catches. This ensures recreational fishing is for leisure only, not for profit, and does not cause significant damage to the ecological environment.

In February 2026, researchers from the Institute of Hydrobiology of the Chinese Academy of Sciences (CAS), Hunan University of Science and Technology, and other institutions published a paper in *Science* [1]. Based on continuous monitoring data from 57 river sections of the Yangtze River mainstream from 2018 to 2023, the paper systematically assessed the ecological response in the early stage of the fishing ban. The results show that since the beginning of the ban, large fish in the Yangtze River have become more abundant, species diversity has rebounded, and the protection and recovery of rare and endemic species have achieved remarkable results.

Professor Yushun Chen (陈宇顺), corresponding author of the paper, said in an interview with *National Science Review* (NSR): ‘The early-stage results of the 10-year fishing ban are very exciting. The rebound of various indicators is basically consistent with our expectations. The reappearance of rare species such as tube fish (*Ochetobius elongatus*) after years of “disappearance” is an unexpected surprise and very encouraging.’

It is worth noting that the 10-year fishing ban is not an isolated plan, but an important part of the ‘Great Protection of the Yangtze River’ proposed by China’s President Jinping Xi (习近平) in 2016, as well as a concrete example of the ‘Beautiful China Initiative’ over the past decade.

## 10-YEAR FISHING BAN: SAVING THE MOTHER RIVER FROM ‘FISHLESS’ CRISIS

Originating from the Tanggula Mountains on the Qinghai-Xizang Plateau, the Yangtze River flows eastward through >10 provinces before emptying into the East China Sea. With a total length of nearly 6400 km, it is the third longest river in the world, after the Nile in Africa and the Amazon in South America. The Yangtze River is also the cradle of the Chinese civilization, with a drainage area of 1.8 million square kilometers, accounting for about one-fifth of China’s total land area.

The Yangtze River provides diverse resources for China: abundant water resources nourish agriculture and industry; the >2800-km ‘Golden Waterway’ runs through east and west; and various water conservancy projects supply a steady stream of electricity. Meanwhile, >400 species of freshwater fish in the Yangtze River, especially large fish such as the four major domestic carps, are important protein sources for riverside residents.

However, with the explosive growth of China’s population over the past few decades, the intensity of human fishing in the Yangtze River has rapidly exceeded the natural recovery capacity of the ecosystem. Since clear records began in 1954, the fishery catch of the Yangtze River has gradually declined, and dropped sharply from the 1970s.

Professor Qiwei Wei (危起伟) from the Yangtze River Fisheries Research Institute of the Chinese Academy of Fishery Sciences (CAFS) said in an interview with NSR: ‘Overfishing is the main cause of the decline of fishery resources in the Yangtze River. My students and I conducted two rounds of questionnaire surveys among fishermen in the Yangtze River mainstream around 2007 and 2013. The results showed that there were few professional fishermen left. In 2013, only 100 valid questionnaires from professional fishermen were collected from below the Gezhouba Dam to the Yangtze Estuary. The questionnaires indicated that most areas of the Yangtze River mainstream had no fish to catch, and many professional fishermen could hardly make a living.’

Professor Ping Zhuang (庄平) from the East China Sea Fisheries Research Institute of the CAFS also told NSR: ‘Before the fishing ban, some fishery resources were already on the verge of depletion and collapse, and some species had disappeared forever. This is directly related to excessive human development and utilization.’

Zhuang pointed out that the decline of riverine aquatic biological resources has multiple causes, five of which are the most prominent: overexploitation of resources (including overfishing), habitat destruction, water pollution, global climate change, and biological invasion.

**Figure 1.**
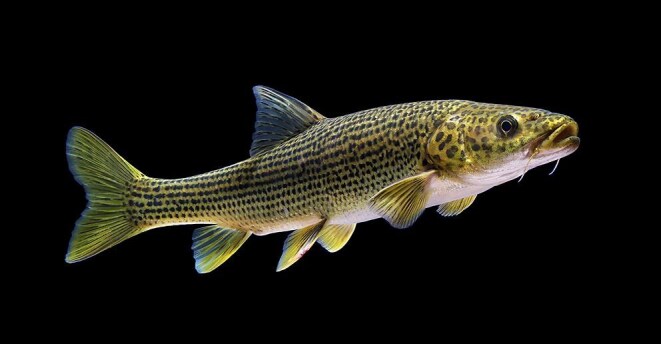
The Jinsha perch (*Percocypris pingi*), a fish species endemic to the Jinsha River in the upper Yangtze basin, is classified as a Class II protected wild animal in China. Since the beginning of the 10-year fishing ban, its population has recovered remarkably. Photo provided by Prof. Ping Zhuang.

All five factors coexist in the Yangtze River. Therefore, the recovery requires joint efforts from multiple aspects. Among them, the 10-year fishing ban directly targets the first cause—overfishing.

Regional and seasonal fishing bans in the Yangtze River have a long history. Initially, seasonal fishing bans were implemented in some river sections to save the Chinese shad, which was on the verge of extinction due to being unable to complete migration after the construction of hydropower stations. Since 2002, the spring fishing ban of the Yangtze River was officially launched, and gradually extended from 3 months to 4 months.

At the Forum on the Conservation of Biological Resources in the Yangtze River held in Shanghai in 2008, CAS academician Wenxuan Cao (曹文宣, born in 1934, one of the pioneers of fish ecology and resource conservation research in China), together with 16 other academicians, jointly proposed the 10-year fishing ban in the Yangtze River. However, this proposal was not immediately supported. Instead, it was questioned and ignored for a long time.

It was not until 2016, when President Xi issued the directive ‘to step up conservation of the Yangtze River and stop its over development’, that a turning point came. In 2018, full fishing bans were first implemented in river sections such as the Chishui River, a tributary of the upper Yangtze River, and finally, the 10-year fishing ban was officially launched across the Yangtze River in 2021.

## EFFECTS OF THE FISHING BAN: DIFFERENT EFFECTS ON THE TWO ‘STAR SPECIES’

Chen and collaborators’ *Science* paper scientifically confirms for the first time that the full fishing ban policy has effectively reversed the 70-year-long decline of fish resources in the Yangtze River. Core indicators such as fish biomass (total weight), species diversity, and fish body condition in the Yangtze River have improved significantly. The biomass of large fish has increased substantially, and the populations of some endangered fish have shown initial recovery trends.

The changes are not only reflected in data: recreational anglers have begun to catch large fish weighing dozens of kilograms, and scenes of Yangtze finless porpoises leaping out of the water have been captured by many people.

Although fish in the Yangtze River have become significantly larger and heavier, the total number of fish has not increased notably. The main reason is that large fish in the Yangtze River usually take 3–5 years to reach sexual maturity, and the current duration of the ban is insufficient to support their reproduction and population recovery. Chen said: ‘We are still in the early stage of recovery. In the future, large fish may grow even bigger, while the total number is unlikely to rise sharply in the short term. It needs to go through a dynamic recovery process. Overall, as long as other stressors are effectively controlled during the fishing ban, the recovery trend of most aquatic organisms is predictable and should be positive.’

The most high-profile ‘star species’ in the Yangtze River are undoubtedly the Yangtze finless porpoise (*Neophocaena asiaeorientalis*) and the Chinese sturgeon (*Acipenser sinensis*), but the fishing ban has had vastly different effects on their population recovery.

**Figure 2.**
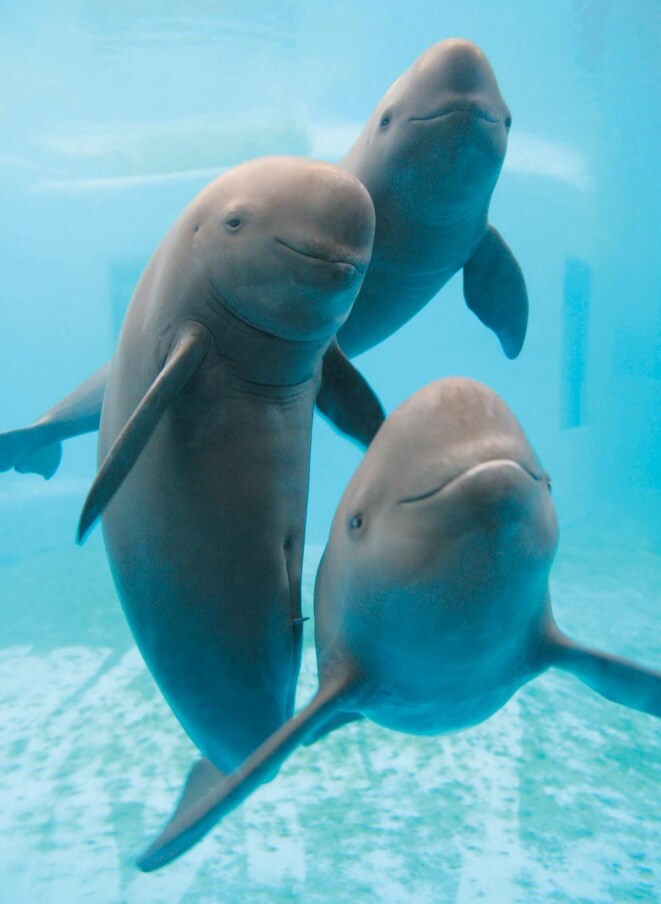
Yangtze finless porpoise in the Institute of Hydrobiology, Chinese Academy of Sciences, on 10 November 2006. Author: Huangdan2060. CC-BY-3.0.

The Yangtze finless porpoise population has rebounded significantly. In the 1990s, the population was ∼2700, and dropped to 1012 in 2017. After the implementation of the fishing ban, in 2022, the population stopped declining and rose to 1249. By 2025, the number has steadily recovered to 1426 [2]. Scenes of finless porpoises playing in the water have reappeared in many water areas such as the Wuhan and Nanjing sections of the Yangtze River.

In sharp contrast, the recovery of the wild Chinese sturgeon population remains elusive. Zhuang said: ‘The reason lies in the different causes of their endangerment. The Yangtze finless porpoise was endangered mainly due to food shortage caused by overfishing, so it has become the direct beneficiary after the fishing ban. In contrast, the Chinese sturgeon is endangered mainly due to habitat destruction, especially the damage to its spawning grounds caused by hydropower projects, which the fishing ban cannot directly solve.’

The Chinese sturgeon is a large anadromous carnivorous fish. It is born in the Yangtze River, migrates to the East China Sea to grow, and when sexually mature, swims upstream along the Yangtze River to spawn in the Jinsha River basin >3000 km from the Yangtze Estuary.

In 1981, the Gezhouba Dam, 1760 kilometers up from the Yangtze Estuary, was officially closed, cutting off the upstream breeding migration route of species such as the Chinese sturgeon and Chinese paddlefish (*Psephurus gladius*). The Chinese paddlefish never reproduced naturally since then and was officially declared extinct in 2019. The Chinese sturgeon was relatively fortunate: it found a small new spawning ground downstream from the Gezhouba Dam and sustained small-scale natural reproduction from 1982 to 2013. However, starting from 2013, due to changes in hydrological and ecological conditions, the last natural spawning ground of the Chinese sturgeon was lost. To this day, it has not reproduced naturally for nine consecutive years.

Artificial breeding and release of juvenile Chinese sturgeon into the Yangtze River have been ongoing for >40 years, with 1.05 million individuals released in 2025. Wei said: ‘Before 2020, I believed that after the fishing ban, the number of mature Chinese sturgeon returning to below the Gezhouba Dam would increase. But there has been no obvious rebound so far, indicating that the reserve of Chinese sturgeon in the ocean has not improved significantly since the fishing ban. The recovery of the wild Chinese sturgeon population will be a long process.’ He believes that saving the Chinese sturgeon requires more than fishing bans and continuous releases; it also involves various efforts, including attempts to reconstruct its spawning grounds, and regulate the fishing in the continental shelf waters of the East China Sea, the Yellow Sea and the Bohai Sea.

Zhuang also recognizes the difficulty of restoring the natural Chinese sturgeon population: ‘Without extraordinary and subversive measures, the spawning grounds may never recover. I am not saying that the Chinese sturgeon cannot be saved, but to save them, humans must give up part of the benefits that we have obtained from hydropower, shipping, urban lighting and so on. We have to return the lost ecological environment to the nature.’

**Figure 3.**
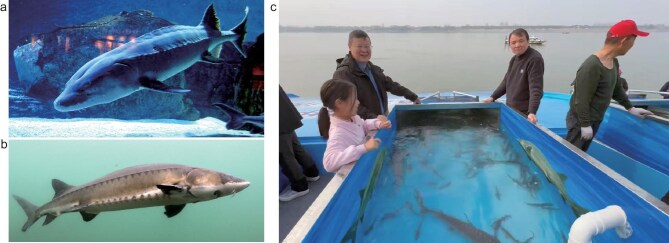
The Chinese sturgeon. (a) A wild Chinese sturgeon, photographed by Prof. Qiwei Wei at the Beijing Aquarium in 2015. (b) An F1 bred Chinese sturgeon, photographed by Prof. Qiwei Wei at the Jingzhou Experimental Base of the Yangtze River Fisheries Research Institute, Chinese Academy of Fishery Sciences, in November 2019. (c) Juvenile bred Chinese sturgeons about to be released into the Yangtze River. Image courtesy of Prof. Qiwei Wei.

Despite the difficulties, Wei remains optimistic: ‘As long as the seed population of artificially bred Chinese sturgeon remains, there is hope. We have already restored the natural populations of the crested ibis and the Milu (Père David’s deer). It is much more difficult for the Chinese sturgeon, but I believe that the recovery will come.’

## INTO THE FUTURE: THE SECOND HALF OF THE ‘GREAT PROTECTION OF THE YANGTZE RIVER’

CAS Academician Jianfang Gui (桂建芳), leader of expert group for the mid-term evaluation of the 10-year fishing ban, said in an interview with NSR: ‘Such a large-scale, full-basin fishing ban is a pioneering initiative of China, and should serve as a global model.’

The entire Yangtze River Basin is within China’s territory, and provinces along the river can obey central orders and sacrifice their own economic interests to implement the full fishing ban. This is almost impossible for international big rivers such as the Rhine and the Danube in Europe. And for the Mississippi River, which lies entirely within the United States, because Americans rarely eat freshwater fish, there is only sporadic commercial fishing and recreational angling on the river. Overfishing is basically not a problem, so no fishing ban measures are needed.

Regarding the second half of the 10-year fishing ban and the ‘Great Protection of the Yangtze River’ after 2030, Gui said: ‘We must build a shared commitment to firmly complete the fishing ban. Ecological restoration requires long-term persistence. In the coming years, we hope more experts will be involved in further monitoring and evaluation to provide data support for the plan after 2030.’

After 2030, some waters of the Yangtze River will likely resume fishery use, while others will remain closed to fishing all year round. The formulation of specific measures will take time.

The fishing ban is only one aspect of the ‘Great Protection of the Yangtze River’. In the future, comprehensive environmental protection and restoration measures are needed. Over the past few years, efforts such as restoring natural shorelines and governing coastal chemical plants in the Yangtze River Basin have been ongoing. Chen said: ‘In addition to enforcing the fishing ban, we must control other stressors, including water quality, shipping, riparian development, industrial and agricultural activities, habitat destruction, sand mining and so on.’

The management of large river ecosystems is an international challenge. Almost all large rivers worldwide have experienced severe pollution and ecosystem damage. Europe and the US developed and faced this challenge earlier than China, and have implemented many conservation measures. Some of their practices, including strict control of industrial emissions, re-naturalization of shorelines, and removal of abandoned dams to restore river connectivity, may offer a useful reference for China.

There are >100 endemic species in the upper Yangtze River. Zhuang said: ‘Although they are not as high-profile as Yangtze finless porpoises and Chinese sturgeon, every species is equally important in the ecosystem. The Yangtze River is a treasure trove of global biodiversity, and every endemic species is valuable and in need of protection.’ For these species, Wei believes it is necessary to verify their population status, formulate targeted conservation measures, and save them from endangerment.

Looking to the future, Zhuang said: ‘The return of tube fish is a miracle. My dream is to see more such miracles, to see more disappeared species reappear in the Yangtze River.’

## Notes

Art editor: Xiaoling Yu.
